# Fibroblast-induced switching to the mesenchymal-like phenotype and PI3K/mTOR signaling protects melanoma cells from BRAF inhibitors

**DOI:** 10.18632/oncotarget.7671

**Published:** 2016-02-24

**Authors:** Kotryna Seip, Karianne G. Fleten, Anna Barkovskaya, Vigdis Nygaard, Mads H. Haugen, Birgit Ø. Engesæter, Gunhild M. Mælandsmo, Lina Prasmickaite

**Affiliations:** ^1^ Dept. Tumor Biology, Oslo University Hospital, The Norwegian Radium Hospital, Oslo, Norway; ^2^ K.G. Jebsen Center for Breast Cancer Research, Institute for Clinical Medicine, Faculty of Medicine, University of Oslo, Oslo, Norway; ^3^ Dept. Pharmacy, Faculty of Health Sciences, University of Tromsø, Tromsø, Norway

**Keywords:** melanoma, BRAF inhibitor, resistance, phenotype switch, fibroblasts

## Abstract

The knowledge on how tumor-associated stroma influences efficacy of anti-cancer therapy just started to emerge. Here we show that lung fibroblasts reduce melanoma sensitivity to the BRAF inhibitor (BRAFi) vemurafenib only if the two cell types are in close proximity. In the presence of fibroblasts, the adjacent melanoma cells acquire de-differentiated mesenchymal-like phenotype. Upon treatment with BRAFi, such melanoma cells maintain high levels of phospho ribosomal protein S6 (pS6), i.e. active mTOR signaling, which is suppressed in the BRAFi sensitive cells without stromal contacts. Inhibitors of PI3K/mTOR in combination with BRAFi eradicate pS6^high^ cell subpopulations and potentiate anti-cancer effects in melanoma protected by the fibroblasts. mTOR and BRAF co-inhibition also delayed the development of early-stage lung metastases *in vivo*. In conclusion, we demonstrate that upon influence from fibroblasts, melanoma cells undergo a phenotype switch to the mesenchymal state, which can support PI3K/mTOR signaling. The lost sensitivity to BRAFi in such cells can be overcome by co-targeting PI3K/mTOR. This knowledge could be explored for designing BRAFi combination therapies aiming to eliminate both stroma-protected and non-protected counterparts of metastases.

## INTRODUCTION

The role of stromal cells in facilitating metastasis and drug-resistance is becoming increasingly recognized [1, 2]. A variety of stromal cells (fibroblasts, endothelial cells and myeloid cells) can reduce cancer cells’ sensitivity to chemotherapy [3–7], and particularly targeted therapy [3]. This effect has been linked to stroma-secreted soluble factors [3, 4, 8], cell-cell junctions [9, 10] or deposition of extracellular matrix (ECM) [11, 12] that can activate pro-survival mechanisms in the cancer cells. Besides, stromal cells can promote epithelial-mesenchymal transition (EMT), a phenomenon when epithelial cancer cells acquire a motile mesenchymal phenotype [13]. It appears that cancer cells with mesenchymal features often show higher resistance to therapies [14, 15]. Thus, phenotype plasticity has been suggested as an important mechanism of drug-resistance and a potential target for therapy [16].

Malignant melanoma is one of the most aggressive, drug-resistant cancer types with a median survival of stage IV patients of 8-18 months [17]. Until recently, effective therapies against metastatic melanoma were lacking. However, significant progress has been achieved with inhibitors targeting frequently mutated BRAF [18]. Mutant BRAF constitutively activates the downstream kinases, MEK and ERK, within the mitogen-activated protein kinase (MAPK) pathway, leading to uncontrolled cell proliferation. BRAF inhibitors (BRAFi), like vemurafenib, suppress MAPK and often induce tumor regression. However, there is variability in the magnitude of the initial response, and resistance usually develops within one year [19]. A number of acquired resistance mechanisms have been disclosed, including re-activation of MAPK [20–22] and activation of alternative signaling pathways, such as the phosphatidylinositol 3-kinase (PI3K) cascade [20, 23]. It has also been observed that melanomas with a mesenchymal gene signature, i.e. reduced expression of melanocytic genes and enhanced expression of mesenchymal genes, are more resistant to BRAFi [24–26]. Furthermore, it has been shown that BRAFi resistance could be promoted by growth factors secreted by stromal cells [3, 8].

Revealing factors affecting sensitivity to BRAFi is important in the search of biomarkers of response, or combination therapies involving BRAFi. It has been shown that BRAFi-induced suppression of a mammalian target of rapamycin complex 1 (mTORC1) is associated with a good BRAFi response, while a maintained mTORC1 activity predicts BRAFi resistance [27]. Typically, mTORC1 is activated *via* the PI3K/AKT pathway [28], but MAPK-dependent activation has also been observed [29, 30]. Since mTORC1 converges both signaling cascades, mTORC1 activity-reflecting substrates could be good indicators of BRAFi response/resistance [27, 30]. One such substrate is phospho ribosomal protein S6 (pS6) that has been proposed as a biomarker for assessing the effectiveness of BRAF-targeted therapies [27, 31].

Here we show that stromal cells, such as lung fibroblasts, reduce melanoma sensitivity to BRAFi and lead to emergence of non-responding cell subpopulations with high levels of pS6. Stroma-mediated protection was dependent on close proximity between the two cell types, which resulted in phenotype switching and signaling re-wiring in melanoma. These findings place stromal cells as important contributors to BRAFi resistance and reveal candidates for targeting stroma-protected parts of the tumor.

## RESULTS

### Melanoma cells in mono-cultures show good response to BRAFi

**Figure 1:**
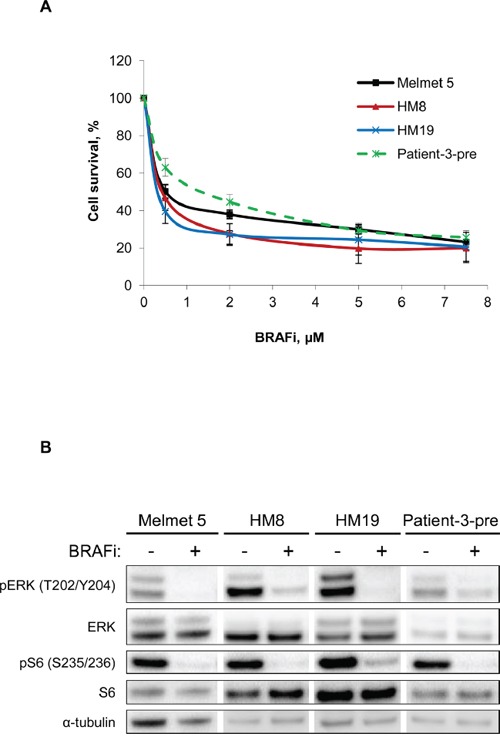
Melanoma cells grown as mono-cultures show good response to BRAFi **A.** Four different melanoma cell lines grown as mono-cultures were treated with different doses of BRAFi for 72 h before the effect on melanoma cells was scored by measuring bioluminescence. The signal intensity in the treated cells was related to the intensity in the non-treated controls and presented in % (average ± SEM, n ≥ 3). **B.** Western blot analysis of the levels of the indicated proteins (α-tubulin, as a loading control) in non-treated or treated (with 1 μM BRAFi for 24 h) melanoma cells.

In this study we applied four BRAF-mutated melanoma cell lines derived from lymph node or brain metastases and stably labeled with GFP-luciferase (further referred as Luc^+^). Cell sensitivity to the BRAF inhibitor vemurafenib was scored by measuring bioluminescence generated by viable luciferase-expressing cells. The method was described previously [6] and further validated in our cell system (Supplementary Figure S1). All tested cell lines showed good response to BRAFi, where half-maximal effective concentrations (EC_50_) were below 1μM (Figure 1A). At the molecular level, we observed a decrease in phosphorylation of ERK and S6 (Figure 1B), markers of the MAPK and mTORC1 activity, respectively. Altogether, this indicates that the four melanoma cell lines, when grown as mono-cultures, are highly sensitive to BRAFi.

### Stromal cells protect melanoma cells from BRAFi *via* proximity-dependent interactions

**Figure 2:**
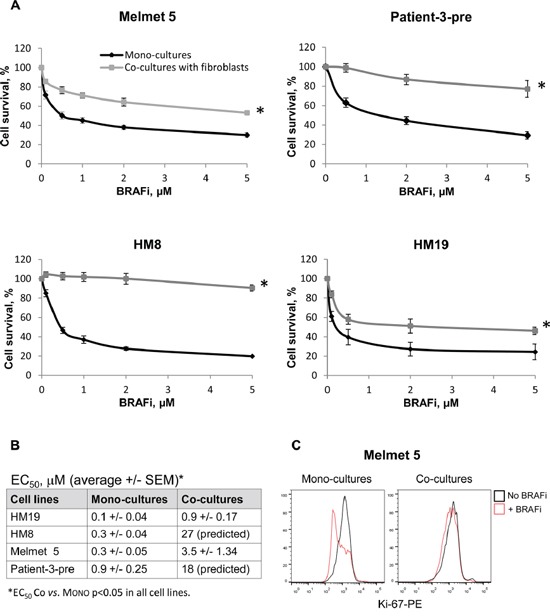
Melanoma cells co-cultured with lung fibroblasts are more resistant to BRAFi **A.** Four different melanoma cell lines were grown as mono-cultures or co-cultures with lung fibroblasts WI-38 with/without BRAFi treatment for 72 h. The effect on melanoma cells was scored by measuring bioluminescence and is presented as % relative to the respective non-treated controls (average ± SEM, n ≥ 3); *, p ≤ 0.05 at all doses (unpaired t-test). **B.** BRAFi EC_50_ values for each cell line treated in the mono-culture or co-culture conditions. **C.** The level of Ki-67 (detected by flow cytometry) in melanoma cells grown as mono-cultures or co-cultures and treated with 1 μM BRAFi for 72 h. The levels in the non-treated controls are shown for comparison.

To evaluate stromal influence on melanoma response to BRAFi, the Luc^+^ melanoma cells were grown together with Luc^−^ lung fibroblasts WI-38 as co-cultures, where the cells are in close proximity to each other. The response to BRAFi was evaluated by measuring bioluminescence produced exclusively by Luc^+^ tumor cells. All four melanoma cell lines showed improved cell survival/growth and significantly increased EC_50_ when treated in the co-culture conditions compared to the mono-culture (Figure 2A, 2B) (no effect on the fibroblasts was observed). In concordance, the level of the proliferation marker Ki-67 stayed high in the treated co-cultures, while it was significantly reduced by BRAFi in the mono-cultures (Figure 2C). Altogether, this indicates that fibroblasts reduce melanoma sensitivity to BRAFi. Since fibroblasts deposit fibronectin, which can diminish BRAFi efficacy [11, 12], we also evaluated melanoma sensitivity to BRAFi on the fibronectin-coated (5μg/cm^2^) surface. Although we observed increased cell survival/growth upon treatment on fibronectin, the protective effect was lower than what was seen in the co-cultures (data not shown). This suggests that adhesion to fibronectin can contribute, but is not the sole mechanism of the fibroblast-mediated protection from BRAFi.

**Figure 3:**
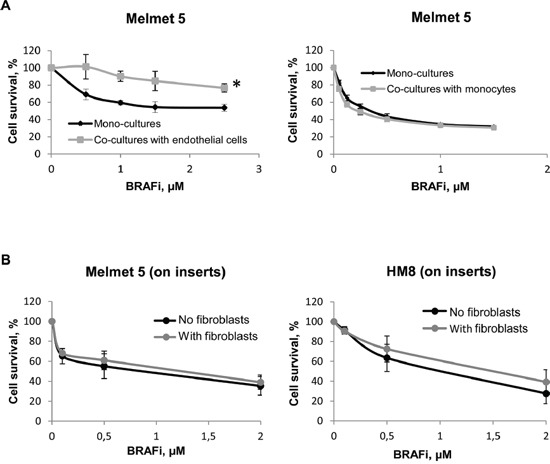
Melanoma cell sensitivity to BRAFi in the presence of endothelial cells, monocytes or fibroblasts separated by a semi-permeable membrane **A.** Melanoma cells were grown either as mono-cultures or co-cultures with endothelial cells HUVEC or monocytes THP-1. **B.** Melanoma cells were grown on semi-permeable inserts, where the fibroblasts or the respective melanoma cells (“No fibroblasts”= Controls) were grown in the bottom chamber. Cells were treated with different doses of BRAFi for 72 h before the drug effect was scored by bioluminescence (A) or the MTS (B) method. The effect on melanoma cells is presented as % relative to the respective non-treated controls (average ± SEM, n ≥ 3); *, p ≤ 0.05 at doses ≥ 1 μM (unpaired t-test).

To examine the influence of other types of stromal cells, we co-cultured Melmet 5 with endothelial cells HUVEC or monocytes THP-1. Endothelial cells, like fibroblasts, adhered to melanoma cells and elicited protection from BRAFi (Figure 3A, left). No adhesion was observed between melanoma cells and monocytes, and the monocytes did not confer any protective influence (Figure 3A, right).

To validate the importance of the tumor-stromal cell proximity/adhesion for the protection, we generated cultures where the cell proximity was disabled by a semi-permeable membrane. Melanoma cells were grown on semi-permeable inserts, whereas fibroblasts were grown on the bottom of a trans-well chamber. This prevents cell-cell adhesion, but allows communication through soluble factors. In contrast to the co-cultures, we did not observe fibroblast-induced protection from BRAFi in Melmet 5 or HM8 grown on the inserts (Figure 3B).

### Protective stroma alters the molecular phenotype of the melanoma cells

**Figure 4:**
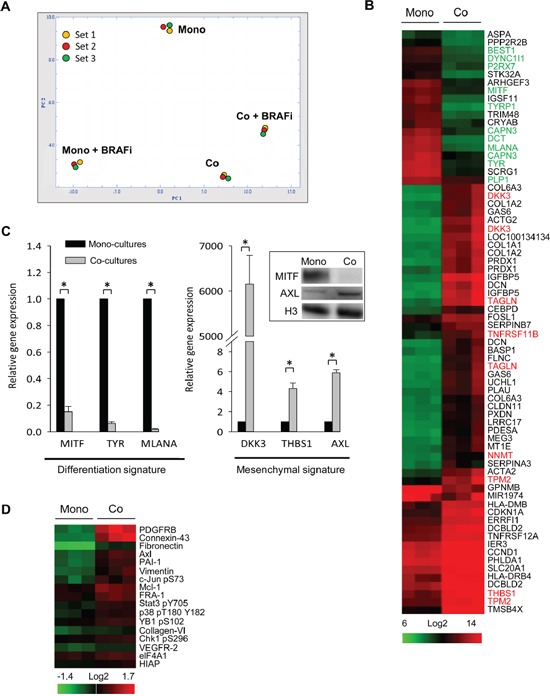
Fibroblasts induce global gene expression changes in melanoma cells Melmet 5 melanoma cells, grown either as mono-cultures or co-cultures with fibroblasts were treated with 1 μM BRAFi for 24 h (controls were not treated), isolated by FACS and analyzed for gene expression (A-C) or protein levels (D). **A.** A PCA plot showing the variance between four groups (3 sets): non-treated mono-cultures (Mono) and co-cultures (Co), and BRAFi-treated mono-cultures (Mono+BRAFi) and co-cultures (Co+BRAFi). **B.** A heatmap showing gene expression levels in melanoma cells from co-*versus* mono-cultures (n=3). The scale bar indicates signal intensity, where green and red colors indicate low and high expression, respectively. Gene names labeled in green denote differentiation signature genes, while genes in red denote invasive/mesenchymal signature genes reported previously [32, 33]. **C.** Relative gene expression of differentiation and mesenchymal signature genes, detected by q-PCR (average ± SEM, n=3); *, p < 0.05. The expression level in the co-cultures was normalized to the level in the mono-cultures set to 1. Insert: MITF and AXL expression changes were confirmed by Western blotting (histone 3 (H3) as a loading control). **D.** A heatmap from the RPPA analysis that shows significantly enriched (at least by 20%) proteins in the melanoma cells from the co-cultures *versus* the mono-cultures (n=3). Proteins are ranked based on fold-enrichment, with the most enriched at the top.

To investigate changes in the molecular profile of melanoma, GFP^+^ Melmet 5 cells from mono-cultures and co-cultures with GFP^−^ fibroblasts, with/without BRAFi treatment, were separated by fluorescence-activated cell sorting (FACS) and analyzed for global gene expression. Principle component analysis (PCA) on normalized data projects variance between the four sample groups. It shows much greater expression differences induced by BRAFi in the mono-cultures than the co-cultures (Figure 4A): 742 *versus* 34 modulated genes, respectively. This indicates a dampened transcriptional response to BRAFi in the melanoma cells in the co-cultures.

Also in the absence of the drug, a clear gene expression variance (608 differentially expressed genes) was observed between the melanoma cells from the co-cultures *versus* the mono-cultures (Figure 4A). This indicates a significantly altered transcriptional profile of melanoma cells due to contact with fibroblasts. The transcriptional signatures of the untreated Melmet 5 cells isolated from the co-cultures *versus* the mono-cultures are compared in Figure 4B. A cluster of the down-regulated genes harbors a number of melanocyte differentiation genes controlled by the master regulator of the lineage, microphthalmia-associated transcription factor MITF. In the up-regulated gene cluster we found genes characteristic for the mesenchymal/invasive phenotype defined previously by others [32, 33]. q-PCR was performed to validate the fibroblast-induced transcriptional changes in selected differentiation genes (MITF, TYR and MLANA), and the mesenchymal signature genes (AXL, THBS1, DKK3) in Melmet 5 (Figure 4C) and HM8 (Supplementary Figure S2A). No significant changes in the expression of all these genes were observed in melanoma cells separated from the fibroblasts by semi-permeable inserts (Supplementary Figure S2B and S2C).

To reveal alterations in the proteome, the FACS-isolated melanoma cells were analyzed by reverse-phase protein array (RPPA). Like the transcriptome, the proteome was significantly altered in the untreated melanoma cells from the co-cultures compared to the mono-cultures. Figure 4D shows the proteins that were most up-regulated in Melmet 5 cells from the co-cultures, confirming an enrichment for Axl and other mesenchymal proteins, such as PDGFRB, fibronectin and vimentin. The same proteins were found to be enriched also in HM8 cells from the co-cultures (data not shown). To note, the purity of the melanoma fractions was carefully validated (see Materials and Methods) to exclude a possibility that the observed mesenchymal signature is due to contamination with fibroblasts. In summary, gene/protein profiling revealed that in the presence of adjacent fibroblasts, the melanoma cells undergo phenotype switching to the de-differentiated mesenchymal state.

### Protective stroma enables BRAFi-treated melanoma to maintain high levels of pS6 and cell cycle progression

**Figure 5:**
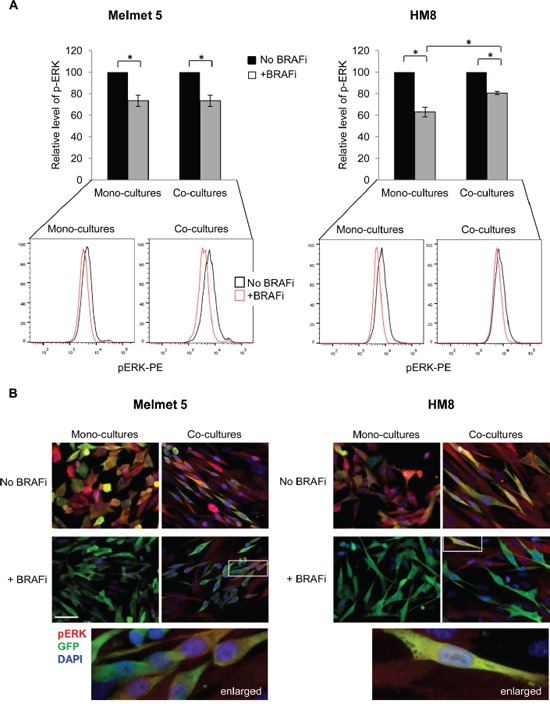
The level of pERK in BRAFi treated melanoma cells from mono-cultures or co-cultures with fibroblasts Melanoma cells were grown as mono-cultures or co-cultures with fibroblasts and treated with 1 μM BRAFi for 24 h (controls were not treated) before analysis for pERK by flow cytometry (A) or immunofluorescence (B). **A.** pERK median level in treated melanoma cells relative to the level in the respective non-treated controls (set to 100) is shown. Data indicates average ± SEM, n≥3; *, p< 0.05 (unpaired t-test). Lower panel: representative histograms indicating pERK levels in single melanoma cells. **B.** The cultures were immunostained for pERK (red) and GFP (green); cell nuclei were stained with DAPI. Only overlay pictures are shown (enlarged region indicated). Scale bar, 50 μm.

As reported previously, low levels of MITF and high levels of AXL may predict melanoma resistance to BRAFi [24, 26], and the resistance may involve (re)activation of MAPK [20, 22] or PI3K/mTOR signaling [23, 27]. To assess MAPK activity, the melanoma cells from the mono-cultures and the co-cultures were analyzed for pERK by flow cytometry. We detected BRAFi-induced suppression of pERK in both culture conditions (Figure 5A). The same was seen by immunofluorescence, where after BRAFi we observed green melanoma cells with low signal of pERK in red (Figure 5B). In the treated co-cultures, though, some single melanoma cells retained ERK phosphorylation (seen as yellow, Figure 5B). In conclusion, generally, we observed pERK suppression by BRAFi in both the mono-cultures and the co-cultures. However, more sensitive methods are needed to quantify whether there is a difference in the suppression magnitude.

**Figure 6:**
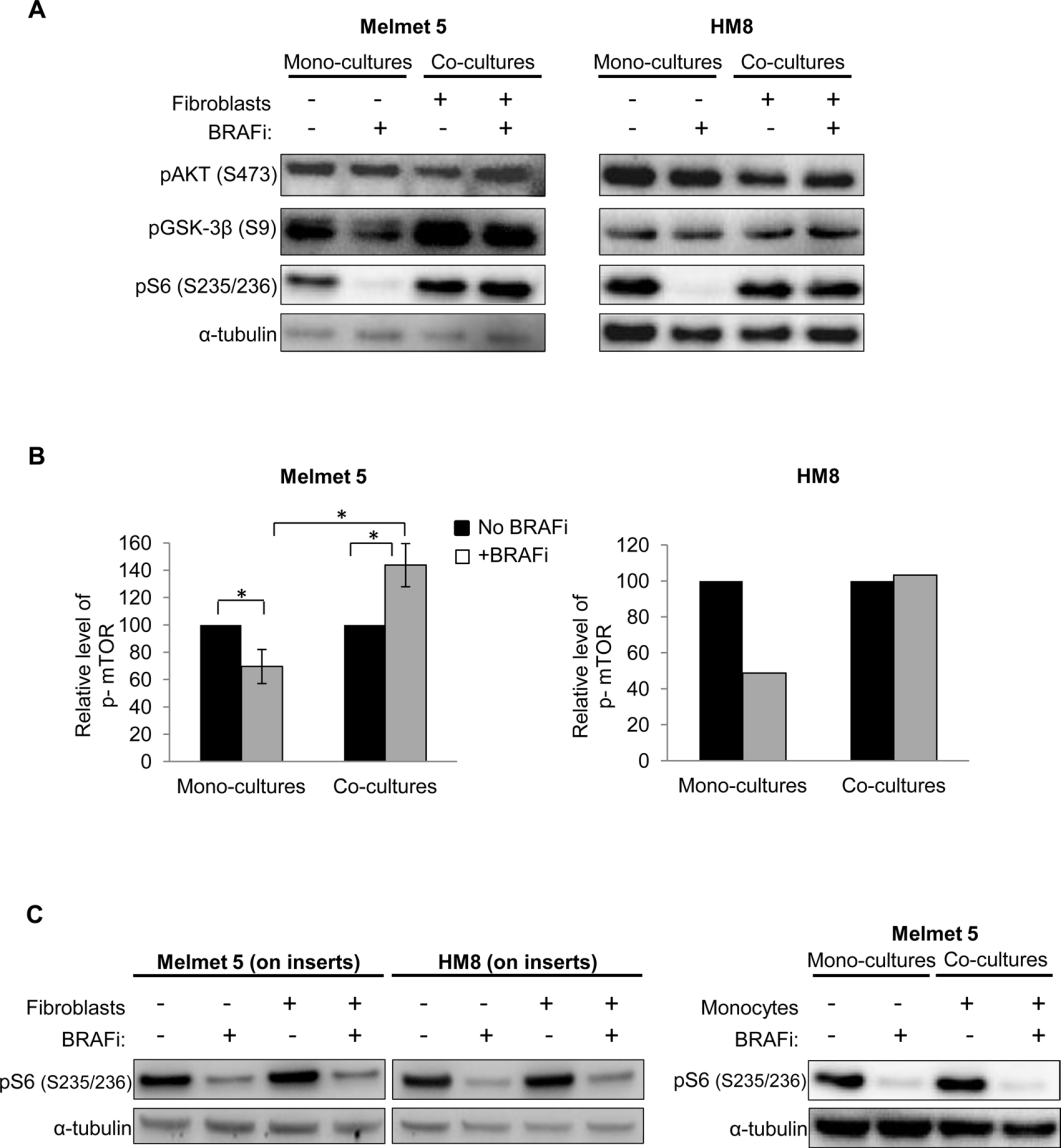
The level of PI3K/AKT/mTOR signaling-associated proteins in BRAFi-treated melanoma cells with/without stromal cells present **A, B.** Melanoma cells were grown as mono-cultures or co-cultures with fibroblasts, treated with 1 μM BRAFi for 24 h (controls were not treated), isolated by FACS and analyzed for the indicated proteins by Western blotting (α-tubulin as a loading control) (A) or automated Simple Western (B). (B) p-mTOR level (after normalization to the loading control, GAPDH) in the treated cells relative to the respective non-treated controls (set to 100). Average ± SEM (n=3 for Melmet 5; n=1 for HM8); *, p< 0.05 by unpaired t-test. **C.** Western blot analysis of pS6 in melanoma cells grown/treated on semi-permeable inserts with/without fibroblasts in the bottom chamber or as co-cultures in the presence of monocytes THP-1.

To compare levels of PI3K/AKT/mTOR-associated proteins in the mono-cultures and the co-cultures, phosphorylated AKT, GSK-3*β*, S6 and mTOR were analyzed in FACS-separated melanoma cells by Western blotting and/or Simple Western immunoassay. We observed only minor alterations in pAKT levels. However, the phosphorylation of an AKT substrate, GSK-3β, and particularly an mTOR substrate, S6, as well as mTOR itself was significantly reduced by BRAFi in the mono-cultures but not in the co-cultures with fibroblasts (Figure 6A, 6B and Supplementary Figure S3A-C). To note, S6 phosphorylation at positions S235/236 (that can be regulated by MAPK in addition to mTOR) and S240/244 (regulated predominantly by mTOR [34]) showed the same pattern of changes (Supplementary Figure S4). Taken together, this indicates a lack of mTOR suppression by BRAFi in the co-cultures. To note, when melanoma-fibroblast contact was prevented by semi-permeable inserts, or when melanoma cells were co-cultured together with monocytes, the level of pS6 was significantly reduced by BRAFi (Figure 6C). This supports the importance of tumor-stroma proximity for sustaining mTOR activity in BRAFi-treated melanoma.

**Figure 7:**
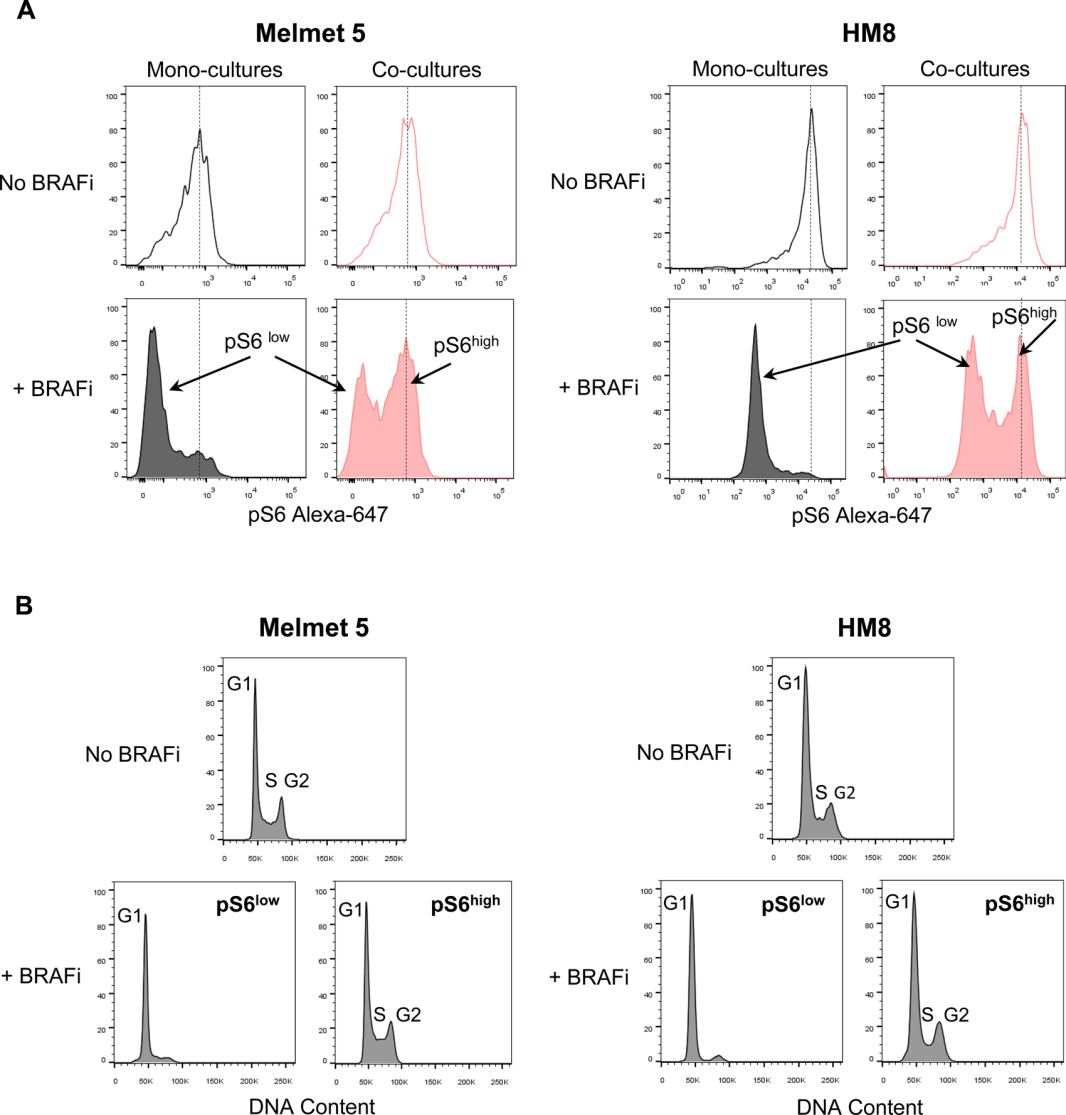
Flow cytometric analysis of pS6 levels and cell cycle in melanoma cells from mono-cultures and co-cultures with fibroblasts Melanoma cells were grown as mono-cultures or co-cultures with fibroblasts, treated with 1 μM BRAFi for 24 h (controls were not treated) before the total cell suspension was analyzed for pS6 levels (A) and cell cycle (B). **A.** Representative histograms indicating pS6 levels in single melanoma cells; dotted lines designate the histogram peak position in the non-treated cells. **B.** DNA content (i.e. cell cycle distribution) in non-treated and BRAFi treated melanoma cells from the co-cultures, where the latter were divided into subpopulations with low (pS6^low^) and high (pS6^high^) levels of pS6.

To explore pS6 levels in single melanoma cells, we employed flow cytometry. The data confirms a BRAFi-induced decrease in pS6 in mono-cultures, which was shown for different drug doses, cell densities or treatment durations (Supplementary Figure S5A-C). In contrast, in the co-cultures with fibroblasts, the level of pS6 remained significantly higher (Figure 7A) and stayed so for the whole treatment duration for up to 3 days (Supplementary Figure S5C). Importantly, in the treated co-cultures we discriminated two melanoma cell subpopulations: those with a reduced pS6 level, and those with high levels, like in controls (Figure 7A). These two subpopulations had a clearly different cell cycle profile: pS6^low^ cells were arrested in G1, while pS6^high^ cells mimicked the untreated controls with cell cycle distribution in G1/S/G2 (Figure 7B). This indicates that the pS6 level discriminates BRAFi-responders that stop progression through a cell cycle, from non-responders that continue cycling.

**Figure 8:**
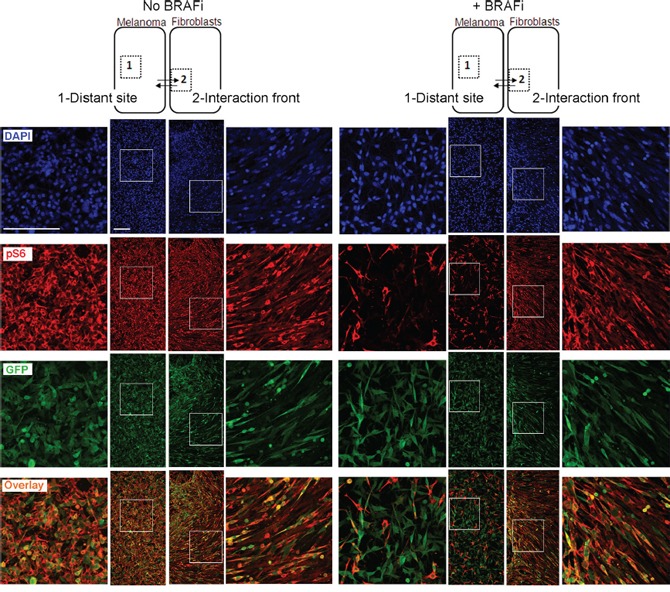
Immunofluorescence analysis of pS6 in asymmetric co-cultures HM8 melanoma cells and fibroblasts were first cultured within adjacent compartments and subsequently allowed to interact (illustrated by arrows), forming asymmetric co-cultures before treatment with 1 μM BRAFi for 24 h (controls were not treated). The cultures were immunostained for pS6 (red) and GFP (green); cell nuclei were stained with DAPI. The staining patterns for the areas representing the distant site (labeled as “1”) and interaction front” (labeled as “2”) are shown. Scale bar, 200 μm.

To verify the stromal role in maintenance of pS6 after BRAFi, we analyzed asymmetric co-cultures, where melanoma culture regions and fibroblast culture regions were allowed to invade each other, forming an interaction front as illustrated in Figure 8. In the interaction front, the green melanoma cells displayed an elongated mesenchymal morphology which was not observed in the melanoma cells at the distant site (Figure 8, GFP). The level of pS6 in the melanoma cells in the interaction front *versus* the distant sites was compared. In the absence of BRAFi, all melanoma cells, regardless of their localization, had high levels of red-stained pS6 (seen as orange cells in the overlay picture). In the presence of BRAFi, the melanoma cells in the interaction front stayed strongly positive for pS6. In contrast, a large fraction of the melanoma cells in the distant site lost the red pS6 signal and appeared green in the overlay picture. Such heterogeneous behavior was observed in both models, HM8 (Figure 8) and Melmet 5 (data not shown). This confirms that fibroblasts make adjacent melanoma cells acquire mesenchymal features and sustain pS6 levels upon treatment with BRAFi.

### Exploring pS6 in melanoma cells from distinct sites *in vivo*

**Figure 9:**
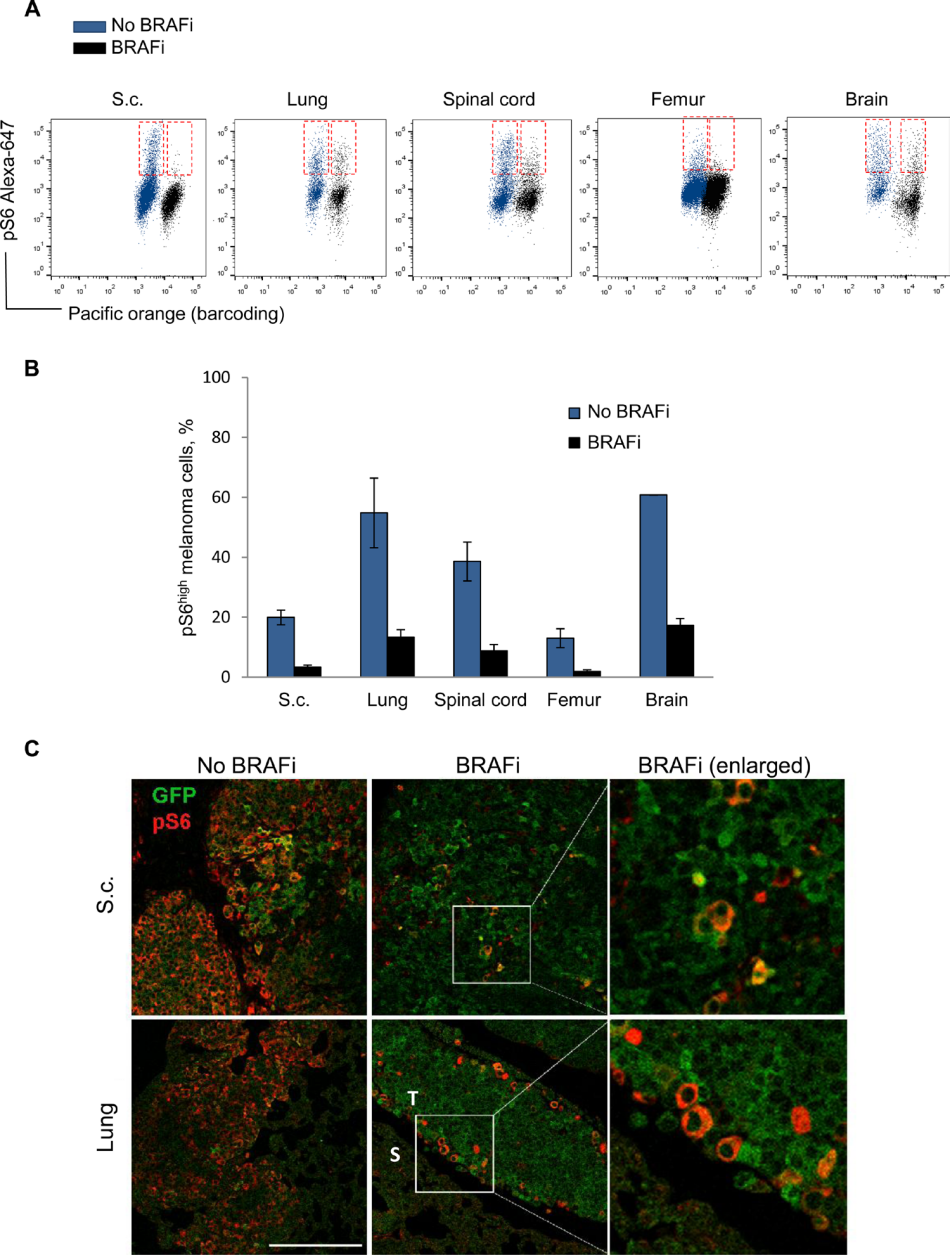
Analysis of pS6 in melanoma cells from distinct sites *in vivo* Melmet 5 melanoma cells were allowed to grow in distinct organs in mice with/without treatment with BRAFi. The metastatic organs and s.c. tumors were collected and analyzed by flow cytometry (A, B) or immunofluorescence (C). **A.** Representative dot-plots showing pS6 levels in Melmet 5 cells from non-treated (blue) and treated (black) animal organs. The pS6^high^ melanoma cell subpopulations are indicated in red boxes. **B.** The bars indicate average percentage of pS6^high^ cells ± SEM (n=3/4), except for the brain samples, where average ± St.Dev. (n=2) is shown. **C.** Immunofluorescence for pS6 (red) and GFP (green) in s.c. tumors (upper panels) and lung metastases (lower panels; S, stromal region; T, tumor region) derived from non-treated and BRAFi treated animals. The pictures show an overlay of pS6 and GFP. Scale bar, 200μm.

The observation that fibroblasts foster melanoma cell subpopulations with sustained pS6 levels, motivated *in vivo* studies where we compared the influence of stroma from different sites. Melmet 5 cells establish tumors in multiple organs when injected into mice through the left ventricle (L.V.), or grown subcutaneously after subcutaneous (s.c.) injection, thereby generating distinct interactions with site-specific stroma. The metastatic cells from different sites of non-treated and BRAFi-treated animals were isolated and analyzed for pS6 by flow cytometry. We observed cellular heterogeneity with respect to the pS6 level, where only a fraction of the melanoma cells were strongly positive for pS6 (pS6^high^) (Figure 9). After BRAFi treatment, the percentage of pS6^high^ cells (Figure 9B) and the mean level of pS6 (Supplementary Figure S6A) was reduced in all sites. Some inter-site variations were observed, where the lung, spinal cord and brain metastases generally harbored a larger fraction of pS6^high^ melanoma cells than e.g. bone metastases or s.c. tumors (Figure 9A, 9B), but this difference has not been further explored. We chose lung metastases and subcutaneous tumors for further analysis by immunofluorescence and confirmed BRAFi-induced reduction in pS6 (Figure 9C). Interestingly, in the treated lung metastases, the remaining pS6^high^ melanoma cells tend to localize at the tumor border next to the stroma (Figure 9C). In the treated s.c. tumors, the distribution of pS6^high^ tumor cells was more random, with less pronounced localization at the tumor-stroma interface (Figure 9C). Collectively, the *in vivo* data indicates that pS6^high^ melanoma cells remain after BRAFi treatment in different metastatic sites, and e.g. in the lung they tend to localize close to the stroma.

### Inhibitors of mTOR or PI3K reduces the protective influence of the fibroblasts *in vitro*

**Figure 10:**
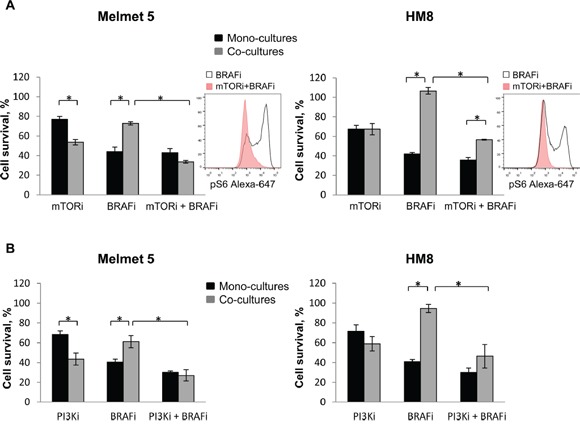
mTOR or PI3K inhibitor reduces the protective influence of fibroblasts in the co-cultures Melanoma cells were grown as mono-cultures or co-cultures with fibroblasts and treated with 0.5 μM BRAFi (A, B), 5 nM mTORi **(A)**, 10 μM PI3Ki **(B)** or the combination as indicated (A, B). The level of pS6 in the co-cultures was analyzed after 24 h by flow cytometry (histogram inserts). Melanoma cell survival/growth was scored after 72 h by measuring bioluminescence and relating the signal in the treated cells to the signal in the non-treated controls (set to 100); average ± SEM (n=3). *, p<0.05 (unpaired t-test).

Based on the pS6 data, we hypothesized that mTORC1, or it activating pathways, might contribute to fibroblast-mediated protection from BRAFi. To test this hypothesis we employed an mTOR inhibitor, everolimus (mTORi) in combination with the BRAFi and compared the response in the mono-cultures *versus* the co-cultures with fibroblasts. We showed that mTORi+BRAFi treatment of the co-cultures eradicates a subpopulation of pS6^high^ melanoma cells (Figure 10A, insert). Consequently, the melanoma cell survival in the co-cultures was significantly reduced after mTORi+BRAFi compared to BRAFi alone. Thus, the survival benefit that we saw in the co-cultures treated with BRAFi alone was eliminated (Melmet 5) or significantly diminished (HM8) after co-administration of mTORi (Figure 10A). A similar potentiation of the drug effect in the co-cultures was observed when BRAFi was combined with a pan-PI3K inhibitor, LY294002 (PI3Ki) (Figure 10B). It should be noted that mTORi/PI3Ki also affected fibroblasts, reducing their number by ∼25% (data not shown). To compensate for this effect, the number of fibroblasts was increased by 25% in the co-cultures where mTORi/PI3Ki was applied. Thereby, we can exclude the possibility that the potentiated effects in the co-cultures might be due to fewer fibroblasts. It is interesting to note that the effect of the single agents, mTORi or PI3Ki, was stronger in the co-cultures than the mono-cultures of Melmet 5, suggesting that the co-cultured cells might be more dependent on PI3K-mTOR signaling.

**Figure 11:**
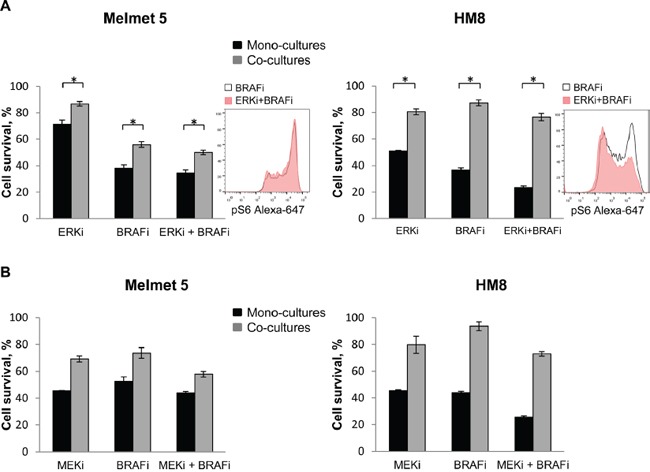
ERK or MEK inhibitor does not eliminate the protective influence of fibroblasts in the co-cultures Melanoma cells were grown as mono-cultures or co-cultures with fibroblasts and treated with 0.5 μM BRAFi (A, B), 10 nM ERKi **(A)**, 0.5 μM MEKi **(B)** or the combination as indicated (A, B). The level of pS6 in the co-cultures was analyzed after 24 h treatment by flow cytometry (histogram inserts). Melanoma cell survival/growth was scored after 72 h by measuring bioluminescence and relating the signal in the treated cells to the signal in the non-treated controls (set to 100). Data indicates average ± SEM (n=5 in A), or ± St. Dev. (from three parallels in a single experiment in B). *, p<0.05 (unpaired t-test).

Re-activation of MAPK was suggested by others to be involved in fibroblast-mediated BRAFi resistance [11], and MAPK can also activate mTORC1 [29]. Therefore, we also tested BRAFi in combination with other inhibitors of the MAPK pathway. In our models, an ERK inhibitor, SCH772984 (ERKi), in combination with BRAFi did not eliminate a subpopulation of pS6^high^ melanoma cell, neither potentiated the treatment effect in the co-cultures (Figure 11A). Likewise, no therapeutic benefit in the co-cultures was observed when BRAFi was combined with a MEK inhibitor (MEKi), MEK162 (Figure 11B). Thus, the co-cultures demonstrated significantly higher cell survival/growth than the mono-cultures after treatment with MEKi or ERKi alone or together with BRAFi.

Collectively, these results indicate that inhibition of PI3K-mTOR, but not other targets within the MAPK-pathway, reduces the protective effect of the stroma and helps to eliminate melanoma cells that become refractory to BRAFi.

### Inhibition of mTOR enhanced the antitumor-effect in BRAFi-treated lung metastases *in vivo*

**Figure 12:**
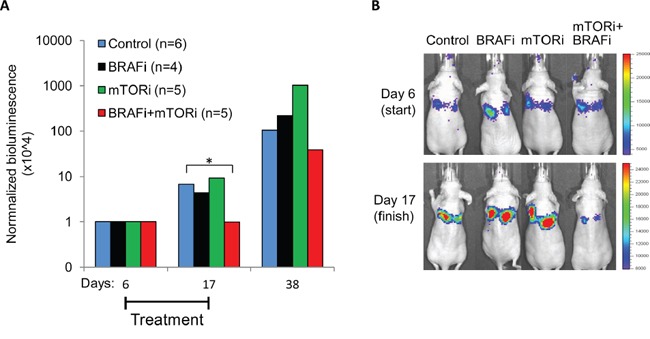
Treatment effect on lung metastases *in vivo* Melmet 5 cells were injected into mice i.v. and were allowed to grow for 6 days before initiation of the treatment with a vehicle (control), 50 mg/kg BRAFi, 5 mg/kg mTORi or a combination of both drugs every weekday until day 17, when the treatment was stopped. Tumor burden was scored at days 6 (start), 17 (finish) and 38 (post-treatment) by live imaging *in vivo*, measuring bioluminescence (p/s/cm^2^/sr) in the IVIS Spectrum instrument. **A.** The bioluminescence signal at days 17 and 38 was normalized to the signal at day 6 (for the non-normalized data, see Supplementary Figure S7). The number of animals per group is indicated in the legend. *, p<0.05 (unpaired t-test). **B.** Representative IVIS pictures showing the animals at the start and the end of the treatment.

Aiming to explore the mTORi+BRAFi combination *in vivo*, we performed a pilot study and showed that by co-treatment with mTORi, we further reduced pS6 levels/pS6^high^ subpopulations compared to BRAFi alone (Supplementary Figure S6B). We concluded that the tested dose of mTORi acts on its molecular target, S6, *in vivo*. In further *in vivo* studies, we compared treatment efficacies on early-stage metastatic lesions established in mouse lung. Melmet 5 cells were delivered to the lung through tail vein injection and were allowed to grow for 6 days before initiating the treatment with a vehicle (control), BRAFi, mTORi or the combination mTORi+ BRAFi. The tumor burden was scored by live imaging *in vivo* tracking bioluminescence. At 6 days post-injection, the metastatic lesions started to be detectable, but were still small (as judged from the bioluminescence, Figure 12B), and presumably rich in tumor-stroma contacts. Exposure of such lesions to the mTORi+BRAFi combination for 12 days inhibited metastatic growth. In contrast, metastases treated with the single agents, BRAFi or mTORi continued growing like the controls (Figure 12A, 12B and Supplementary Figure S7). Although the metastatic growth was resumed after the combined treatment was stopped, the temporal suppression of the tumor development supports the idea that active mTORC1 contributes to cell proliferation in a part of a BRAFi-treated tumor. Thus, co-suppression of mTOR and BRAF activity might be beneficial in early-stage metastases with abundant stromal contacts.

## DISCUSSION

Adaptive and less frequently observed innate resistance to MAPK pathway inhibitors (MAPKi) is a well-known problem in the clinical management of malignant melanoma. In the present study, we have revealed a mechanism of stroma-promoted resistance. Stromal cells, such as lung fibroblast, reduced melanoma sensitivity to BRAF inhibition through proximity-dependent interactions. The protective influence of the stroma might have significant implications for MAPK targeted therapies, since it can lead to emergence of non-responding cancer cell subpopulations in an otherwise well-responding tumor.

Consistent with previous reports [27, 31], we identified one of the mTORC1 substrates, pS6, as a marker discriminating BRAFi responders from non-responders, and demonstrated its association with the protective influence of the stroma. Eradication of pS6^high^ melanoma cells by co-targeting mTOR, potentiated an anti-cancer effect in the BRAFi-treated melanoma models with abundant stromal contacts. Thus, BRAFi-treated cancer cells with sustained pS6 levels might represent subpopulations that got a survival advantage from adjacent stroma. It is tempting to propose that the therapeutic benefit that we see *in vivo* after co-inhibition of BRAF and mTOR is due to co-targeting stroma-independent and stroma-dependent counterparts of metastases. The fact that we observe such a benefit in early-stage metastatic lesions, where stromal contacts are abundant, supports but does not prove this scenario. Although we primarily focused on suppressing mTOR to overcome stromal protection, our *in vitro* data indicates an equally good effect with PI3K inhibitors. Given that in some cases mTOR inhibition can lead to undesirable activation of AKT [23], targeting the upstream kinases, like PI3K, is an attractive alternative. Multiple clinical trials are now initiated to evaluate the benefit of co-inhibition of MAPK and mTOR or PI3K (https://clinicaltrials.gov/ct2/show/NCT01596140) [35]. Their rationale, though, does not lean specifically on stroma-facilitated resistance to MAPKi.

The protective effect of stroma could be explained by stroma-induced phenotype transition in the adjacent melanoma cells. We showed that in the presence of fibroblasts, melanoma cells acquired a de-differentiated mesenchymal-like phenotype. Such a phenotype has been linked to BRAFi resistance in several previous studies, where it was described as a cell-autonomous mechanism of resistance [24, 26, 36]. We show that this phenotype can be induced by extrinsic signals from the stroma. This phenotype displayed reduced sensitivity not only to BRAFi, but also MEKi, ERKi or combinations of these, as was also shown by others, but not in a tumor-stroma context [24, 26]. The fact that resistance was observed even though pERK was suppressed, raised doubts about MAPK dependence in this phenotype [24, 26]. In contrast, the sensitivity to PI3K/mTOR inhibition was preserved or even enhanced, suggesting that the stroma-induced phenotype might prefer PI3K/mTOR signaling. By switching to the mesenchymal phenotype, melanoma cells gained additional “tools” to signal via PI3K, e.g. up-regulated receptor-tyrosine kinases (RTKs) like Axl and PDGFRB or ECM fibronectin [26, 37]. Re-wiring of signaling towards the PI3K pathway was also reported for epithelial cancer cells that undergo EMT and acquire alternative RTKs [38]. In line with our observations, several recent studies showed that stroma-promoted BRAFi resistance involves the PI3K pathway [37, 39]. In contrast, Hirata *et al.* [11] demonstrated stroma-dependent re-activation of ERK as a resistance mechanism, which we could not confirm, since no therapeutic benefit after co-administration of MEKi or ERKi was seen in our co-cultures. Collectively, this favors BRAFi combinations with RTK/PI3K/mTOR inhibitors rather than MEK/ERK inhibitors in situations where a mesenchymal phenotype is promoted, e.g. tumors with abundant stroma. However, more profound analysis on MAPK is needed to clarify whether/how this pathway is implicated in the stroma-induced phenotype.

The majority of previous studies linked stroma-induced BRAFi resistance to stroma-derived growth factors [3, 8, 37], which could act on distant cancer cells. This contradicts our and Hirata et al.'s [11] observations on the importance of close proximity between the tumor and the stromal cells. Based on our data, we propose that a tumor-stroma interaction front might be a site where BRAFi resistant melanoma cells reside, which remains to be validated in clinical material. Adjacent stromal cells could deposit ECM to which melanoma cells could attach and gain a survival advantage, as shown by others [11, 12]. Direct cell-cell communication *via* e.g. gap junctions could be another mechanism for how adjacent cancer cells might gain drug-resistance [9, 10]. Although we observed stroma-induced enrichment for the ECM component fibronectin and a gap junction constituent Connexin-43, we have not yet explored their significance.

In conclusion, we have shown that under the influence of adjacent fibroblasts, melanoma cells acquire a mesenchymal-like phenotype with enhanced resistance to MAPKi. This resistance could be overcome by co-inhibiting PI3K/mTOR signaling. Understanding the link between stroma-induced mesenchymal transition and alterations in signaling should uncover nodes for targeting stroma-protected cancer cells. Thereby, BRAFi combination therapies could be designed to target both stroma-dependent and independent counterparts of metastases.

## MATERIALS AND METHODS

### Cell lines and drugs

Malignant melanoma cell lines: Melmet 5 (derived from lymph node metastases), HM8 and HM19 (both derived from brain metastases), were established from melanoma patients at the Oslo University Hospital, The Norwegian Radium hospital (Oslo, Norway) as described previously [40] (REK No: S-01252; 2.2007.997; 2011/2183). Patient-3-pre cell line (derived from lymph node metastases [41]) was generously provided by Prof. Peter Hersey (University of Sydney, Sydney, Australia). All melanoma cells were transduced with lentivirus, carrying a human ferritin promoter-driven GFP-Luc construct described previously [42] (kindly provided by Dr. Glenn Merlino, NIH, MD). Human lung fibroblasts WI-38 were obtained from the ATCC (Rockville, MD). Human primary umbilical vein endothelial cells (HUVEC) and human monocyte cell line THP-1 were provided by Prof. Guttorm Haraldsen (Oslo University Hospital, Norway; REK S-05152) and Prof. Rigmor Solberg (University of Oslo, Norway), respectively.

All melanoma cell lines were cultured in RPMI 1640 medium, supplemented with 10% fetal calf serum (FCS) and 2 mM L-Alanyl L-Glutamine (all from Sigma-Aldrich, St. Louis, MO). The culture medium for THP-1 was additionally supplemented with 0.05 mM 2-mercaptoethanol (Sigma-Aldrich). WI-38 fibroblasts were cultured in EMEM medium (ATCC, Manassas, VA) supplemented with 10% FCS. HUVECs were cultured in MCDB131 medium (Gibco, Paisley, UK) supplemented with 7.5% FCS, 2 mM L-Alanyl L-Glutamine, 20 mM Hepes, 100 units/ml penicillin and 100 g/ml streptomycin, 1 μg/ml hydrocortisone (all from Sigma-Aldrich), 10 ng/ml EGF (R&D Systems, Minneapolis, MN) and 1 ng/ml bFGF (PeproTech, Rocky Hill, NJ). All cells cultures were maintained at 37°C in a humidified atmosphere containing 5% CO_2_ and were routinely tested for mycoplasma and cell ID.

### Drugs

BRAFi vemurafenib and ERKi SCH772984 were from Selleck Chemicals (Houston, TX), mTORi everolimus was from Novartis (Basel, Switzerland) and Sigma Aldrich, MEKi MEK162 was from MedChemExpress (Monmouth Junction, NJ), PI3Ki LY294002 was from Cell Signaling Technology (Danvers, MA). All drugs were dissolved in DMSO. For animal studies, the drugs were further diluted in 0.5% metylcellulose (Sigma-Aldrich).

### Co-cultures and cultures in trans-well inserts

Co-cultures were prepared by seeding GFP-Luc-labeled melanoma cells together with non-labeled stromal cells (WI-38, THP-1 or HUVEC) in the respective medium, at a ratio 1:4, and a total cell density up to 7 × 10^3^ cells/well in 96-well plates or 75 × 10^4^ cells/T25 flasks. The same cell density was used for the mono-cultures.

To prepare asymmetric co-cultures, the melanoma cells and the fibroblast (20 × 10^3^ each) were seeded in separated compartments within culture-inserts from Ibidi (Martinsried, Germany) placed on glass cover slips. After removing the inserts, the cells were cultured for 4 days allowing them to fill the gap and interact, before BRAFi was applied for 24 hours treatment (no BRAFi for controls).

To prepare cultures in trans-well inserts, 8 × 10^3^ or 20 × 10^4^ melanoma cells were seeded out into 0.4 μm-pore membrane inserts in 24- or 6-well plates (Costar, Corning, NY), respectively. In the bottom wells, either fibroblasts or respective melanoma cells (for controls) were seeded, keeping the insert:bottom cell ratio at ∼1:2.

For gene expression and protein analysis, the co-cultures were pre-incubated for 48 hours followed by 24 hours treatment with 1 μM BRAFi. Melanoma cells were separated from the fibroblasts by FACS, gating on the GFP signal and collecting only clearly GFP positive melanoma cells as shown in Supplementary Figure S8A. The mono-cultures were handled identically. The purity of the FACS-separated melanoma fractions (i.e. no contamination with fibroblasts) is guaranteed since the gating was stringent; no fibroblast-specific (female) transcripts were found in the melanoma (male) fractions; no descent in male-specific transcripts and luciferase (tag on melanoma) mRNA was detected.

### Cell survival assays

Cell survival was evaluated either by measuring luciferase-generated bioluminescence or using the MTS assay. For the bioluminescence method, cells were grown/treated in white 96-well plates (Costar), and before measurement the culture medium was replaced with a fresh medium containing 0.1 mg/mL D-luciferin (Biosynth AG, Staad, Switzerland); after 10 min bioluminescence was measured by a plate reader (Victor^2^ 1420 Multilabel Counter, Perkin Elmer, Waltham, MA). For the MTS method, cells were grown in clear 96-well plates (Falcon, Durham, NC) or 24-well inserts (Costar). Before measurement, 20 μl of CellTiter 96^®^AQueous One solution (Promega, Madison, WI) was applied per each 100 μl medium, followed by ∼1 h at 37°C incubation, and absorbance was measured at 490 nm by the plate reader.

### Flow cytometry

Collected cells (from cultures or disintegrated tumor tissue) were fixed for 15 min in 1.6% paraformaldehyde (PFA) at room temperature (RT) and permeabilized with 100% ice cold methanol. Up to four samples were given a fluorescent “barcode” by adding pacific orange (PO) dye (Life Technologies, Carlsbad, CA) at different concentrations ranging from 0 to 2 ng/μl (see Supplementary Figure S8B). For the analysis of the *in vivo* samples, we also included a “spike” control (Melmet 5 from *in vitro*), which received no PO (see Supplementary Figure S8C). After incubation at RT for 30 min, followed by washing, the barcoded samples were combined for simultaneous staining with antibodies against pS6-Alexa 647 (Cell Signaling Technology, #4851 dilutions 1:80 for *in vitro* and 1:50 for *in vivo* samples), pERK-PE (Cell Signaling Technology, #5315; dilution 1:5) or Ki-67-PE (BD Biosciences, #556027; dilution 1:5) for 30 min at RT. For cell cycle analysis, pS6-Alexa 647 stained cells were additionally stained with 1.5 μg/ml Hoechst 33258 (Life Technologies, #H3569) for 30 min at 37°C. The samples were analyzed on an LSR II flow cytometer (BD Bioscience, San Jose, CA). BD FACS Diva^™^ software was used to control the flow cytometer and Flow Jo software (FlowJo, Ashland, OR) was used to analyze the data.

### Protein analysis

Protein lysates were prepared by re-suspending the cells in lysis buffer (150 mM NaCl, 50 mM Tris pH 7.5, 0.1% Nonidet P40) containing protease- and phosphatase inhibitors (Roche Applied Science, Mannheim, Germany) followed by ultrasonication. For Western blotting, total cellular proteins (20 μg) were separated on NuPAGE^®^ Novex 4-12% Bis-Tris Gel (Life Technologies). After transfer to a polyvinylidene difluoride membrane (Merck Millipore, Billerica, MA), it was blocked either with 10% BSA or 5% dry milk solution and incubated with primary antibodies at 4°C overnight, followed by incubation with secondary antibodies at RT for 1 h. After application of Super Signal West Dura kit solution (Thermo Scientific, Rockford, IL), the membrane was developed in a Syngene instrument (Syngene, Cambridge, UK) using the GeneSnap software.

The Simple Western immunoassay was performed on a PeggySue™ (ProteinSimple, San Jose, CA) applying 0.4 μg total protein lysate and using the Size Separation Master Kit with Split Buffer (12-230 kDa) according to the manufacturer's instructions. The Compass software (ProteinSimple, version 2.7.1) was used to program the PeggySue-machine and for quantification of the results.

The following antibodies from Cell Signaling Technology were used: anti-pS6 (#4858; 1:2000); anti-S6 (#2217; 1:1000); anti-pERK (#4370, 1:2000), anti-ERK (#4695, 1:1000); anti-pAKT (#9271, 1:1000); anti-p-mTOR (#5536, 1:50); anti-pGSK-3β (#9339, 1:50); anti-MITF (#12590, 1:2000), anti-H3 (#4499, 1:2000) and anti-GAPDH (# 5174, 1:200). Mouse anti-AXL (used at 1:4000) was kindly provided by Prof. James Lorens (University of Bergen, Norway). Mouse anti-α-tubulin (Merck Millipore #CP06) was used at a dilution 1:5000.

The RPPA analysis on FACS-separated melanoma cells was performed at MD Anderson RPPA core facility (Houston, TX). In brief, denatured cell protein lysates were arrayed at serial dilutions on nitrocellulose-coated slides. Each slide was probed with a validated primary antibody (specified at the core facility's home page; availbale upon request) followed by a biotin-conjugated secondary antibody; the signal was detected by a DAB colorimetric reaction. The slides were scaned and analyzed using a MicroVigene software (VigeneTech, Carliste, MA) to determine spot intensities. Each dilution curve was fitted with a logistic model (“Supercurve Fitting”). The data were log_2_-transformed and converted into standard scores by subtracting the mean of the whole screen and dividing by the standard deviation of the whole screen (for a given antibody).

### Immunofluorescent staining (IF)

Cells seeded on coverslips were fixed for 15 min in 1.6% PFA at RT and permeabilized either with 100% ice cold methanol or 0.05% Saponin solution. Cells were stained with primary antibodies (rabbit anti-pERK, #4370, 1:200 or rabbit anti-pS6, #2215, 1:200; both Cell Signaling Technology) in combination with goat anti-GFP (ab5450, 1:2000, Abcam (Cambridge, UK)) overnight at 4°C, followed by 1 hour staining with secondary antibodies (donkey anti-rabbit DL549 and donkey anti-goat Alexa 488) at RT and counterstained with DAPI (all from Life Technologies). Fluorescence images were taken using Zeiss LSM confocal microscope (Zeiss, Oberkochen, Germany) or inverse microscope Olympus IX8 (Olympus, Norway) and analyzed with ZEN 2009 Light Edition or Cell^P softwares, respectively.

Sections of paraffin embedded tumor tissue were deparaffinized with xylene and ethanol and boiled in antigen unmasking solution (1:100, Vector laboratories Inc, Burlingame, CA) for 15 min in a microwave. The slides were stained as above, except that anti-GFP was diluted 1:1000.

### RNA isolation and q-PCR

Total RNA was isolated from the FACS-separated cell pellets using TRIzol^®^ reagent (Life Technologies). 1 μg RNA was reverse transcribed using cDNA Synthesis Kit (Quanta Biosciences, Geithersburg, MD). q-PCR reaction was run in duplicates using 25 ng cDNA, mixed with 300 nM of each primer, 200 nM FAM-labeled probe (from the Universal Probe Library collection, Roche Applied Science) and 1x PerfeCTa q-PCR SuperMix (Quanta BioSciences) adjusted up to total volume of 25 μL/well in 96-well plates (Bio-Rad, Hercules, CA). The PCR was performed using Bio-Rad CFX Connect™ Real Time PCR machine (Bio-Rad). Data was analyzed using Bio-Rad CFX Manager software.

### Illumina microarray and data analysis

RNA was amplified from 500 ng total RNA using the Illumina^®^ TotalPrep^™^-96RNA amplification kit (Life Technologies) according to the manufacturer's protocol. For each sample, 750 ng labeled cRNA was hybridized to Illumina Human HT-12v4 Expression BeadChip (Illumina, San Diego, CA). Hybridization was performed by the Norwegian Radium Hospital Microarray Core Facility according to Illumina protocols. The scanning was done with the iScan system. The data were annotated using the HumanHT-12_V4_0_R2_15002873_B.bgx file from Illumina. Sample data were quantile normalized and log_2_-transformed in Illumina GenomeStudio^®^ software. Preprocessed data was imported into J-Express v2012 (www.molmine.com) where a multi-group ANOVA analysis was performed, followed by the application of principal component analysis (PCA) on the top ranked ANOVA-filtered genes to visualize differences between groups. Significance of Microarray (SAM) analysis was applied to identify differently expressed genes. Genes were considered significant if fold change FC ≥ 1.5 and a fold discovery rate FDR ≤ 1%. The microarray data have been deposited in NCBI's Gene Expression Omnibus and are accessible through GEO Series accession number GSE67637 (http://www.ncbi.nlm.nih.gov/geo/query/acc.cgi?acc=GSE67637).

### Animal studies

To analyze pS6 levels, GFP-Luc-labeled Melmet 5 cells were injected into athymic nude foxn1^nu^ mice into the left ventricle (L.V) or subcutaneously (s.c.) (15 × 10^4^ or 1 × 10^6^ cells/animal, respectively). After approximately one month, the animals were randomized before initiation of the treatment by oral gavage with BRAFi vemurafenib (50 mg/kg) or vehicle (DMSO/metylcellulose) twice/day, 5 times in total. Where indicated, mTORi everolimus (5 mg/mL) was applied in addition once/day. Animals were sacrificed 2 hours after the last treatment. Brain, lung, femur, spinal cord and subcutaneous tumors were collected and either disintegrated mechanically for analysis by flow cytometry, or formalin-fixed and paraffin-embedded for IF. For testing the treatment efficacy on early metastatic lesions, 75 × 10^4^ GFP-Luc-labeled Melmet 5 cells were injected intravenously (i.v.) leading primarily to lung metastases. After 6 days, the animals were randomized and treated with either 50 mg/kg BRAFi vemurafenib, 5 mg/kg mTORi everolimus, a combination of both drugs or vehicle every weekday for two weeks. Tumor growth was followed by reading bioluminescence (after intraperitoneally injection of 4mg D-Luciferin in 200 μL PBS) using an *in vivo* imaging system IVIS Spectrum (Perkin Elmer, Waltham, MA).

### Statistical analysis

Two-tailed Student's unpaired t-test was used in all statistical analysis. Differences were considered statistically significant if p-values were equal/below 0.05.

## SUPPLEMENTARY FIGURES


