# Supplementary material for: Fibroblast-induced switching to the mesenchymal-like phenotype and PI3K/mTOR signaling protects melanoma cells from BRAF inhibitors
Source: Oncotarget. 2016 Feb 24;7(15):19997–20015. doi: 10.18632/oncotarget.7671 (PMC4991434; doi:10.18632/oncotarget.7671)
Supplement: Supplementary file 1 [file oncotarget-07-19997-s001.pdf]

SUPPLEMENTARY FIGURES

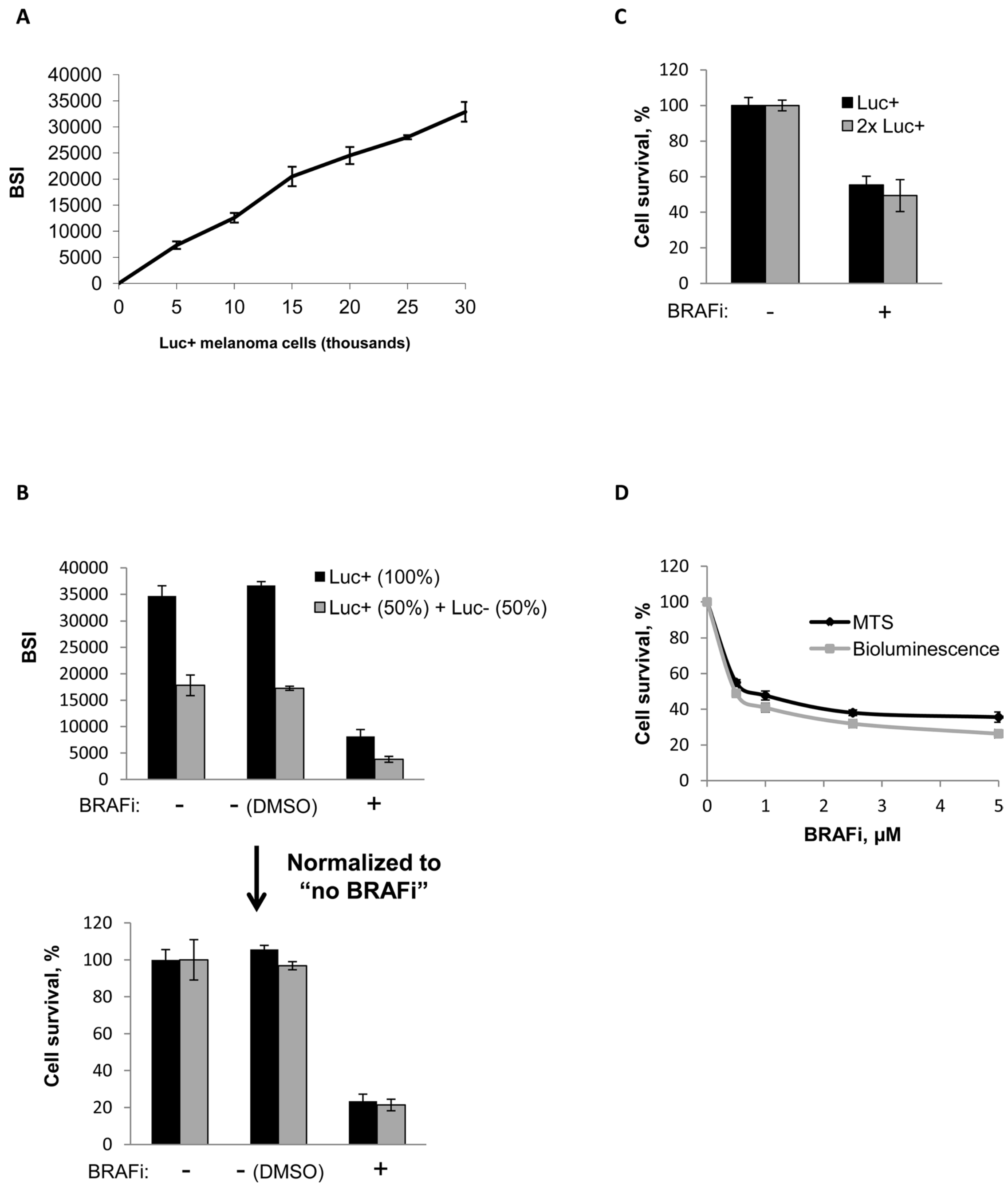

Supplementary Figure S1: Validation of the bioluminescence method for tracking Luc⁺ melanoma cells. A. Linear relationship between cell number and bioluminescence signal intensity (BSI). **B.** Cultures consisting of only Luc⁺ Melmet 5 cells or a mixture of Luc⁺ and Luc⁻ Melmet 5 melanoma cells (ratio 1:1), treated with 5 µM BRAFi for 72 h before BSI was measured. Data indicates average ± St.Dev. from three parallels in a single experiment. **C.** Luc⁺ Melmet 5 melanoma cells cultured at different (2-fold) cell densities while treated with BRAFi. Cell survival was scored by measuring BSI and normalized to the non-treated controls. Data indicates average ± St.Dev. from three parallels in a single experiment. **D.** Comparison of Melmet 5 melanoma cell survival after treatment with different doses of BRAFi, evaluated by measuring BSI and by the standard MTS method. Data indicates average ± SEM (n=3).

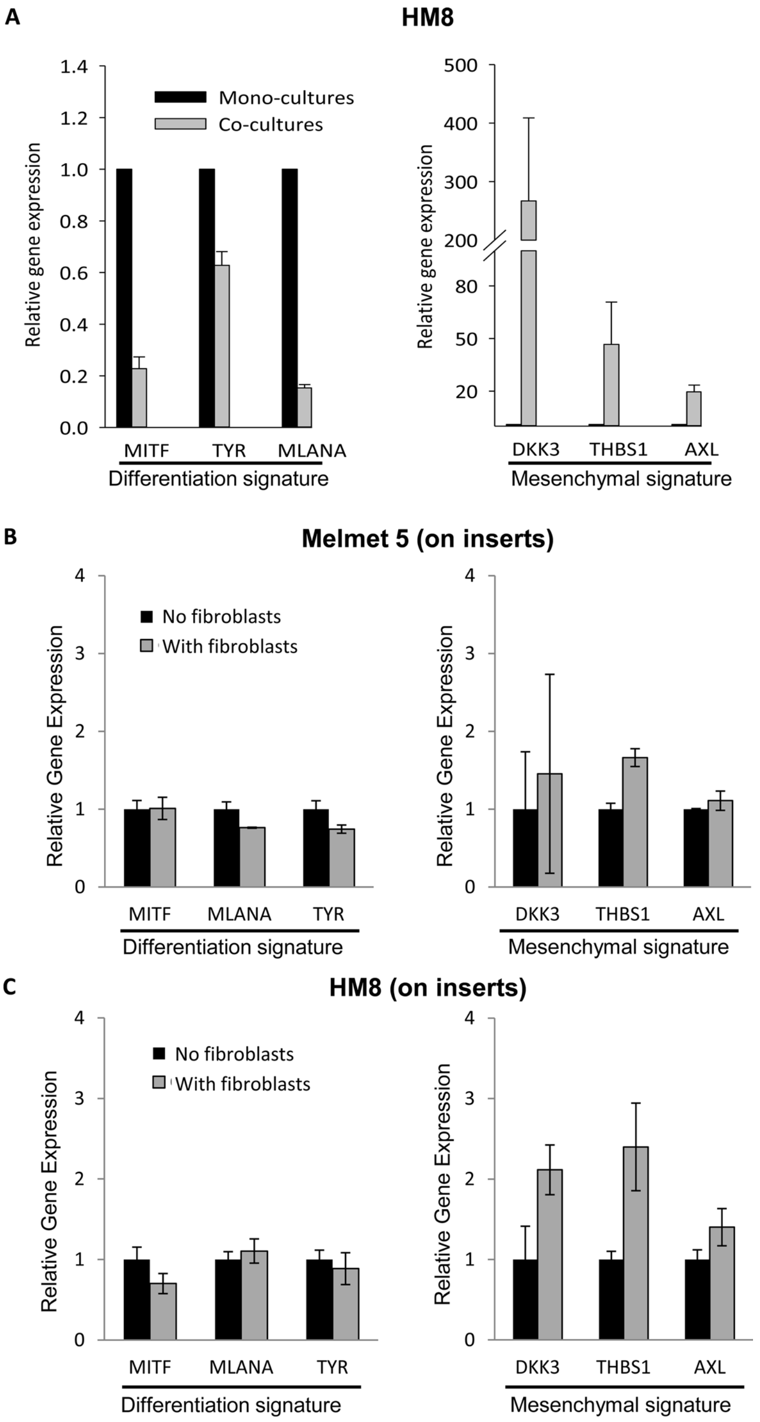

Supplementary Figure S2: Expression of differentiation and mesenchymal signature genes in melanoma cells with/without fibroblasts present. A. HM8 melanoma cells were grown as mono-cultures or co-cultures with fibroblasts, treated with 1 µM BRAFi for 24 h (controls were not treated), isolated by FACS and analyzed for the expression of the indicated genes by q-PCR. **B, C.** Melmet 5 (B) and HM8 (C) melanoma cells were grown on semi-permeable inserts, where fibroblasts or the respective melanoma cells (“No fibroblasts”= Controls) were grown in the bottom chamber, treated as above and analyzed for gene expression. The expression levels in the presence of fibroblasts were normalized to the level in the absence of fibroblasts (which was set to 1). The bars indicate average ± St. Dev. (n=2 in A) and ±St. Dev. (two parallels in a single experiment in B, C).

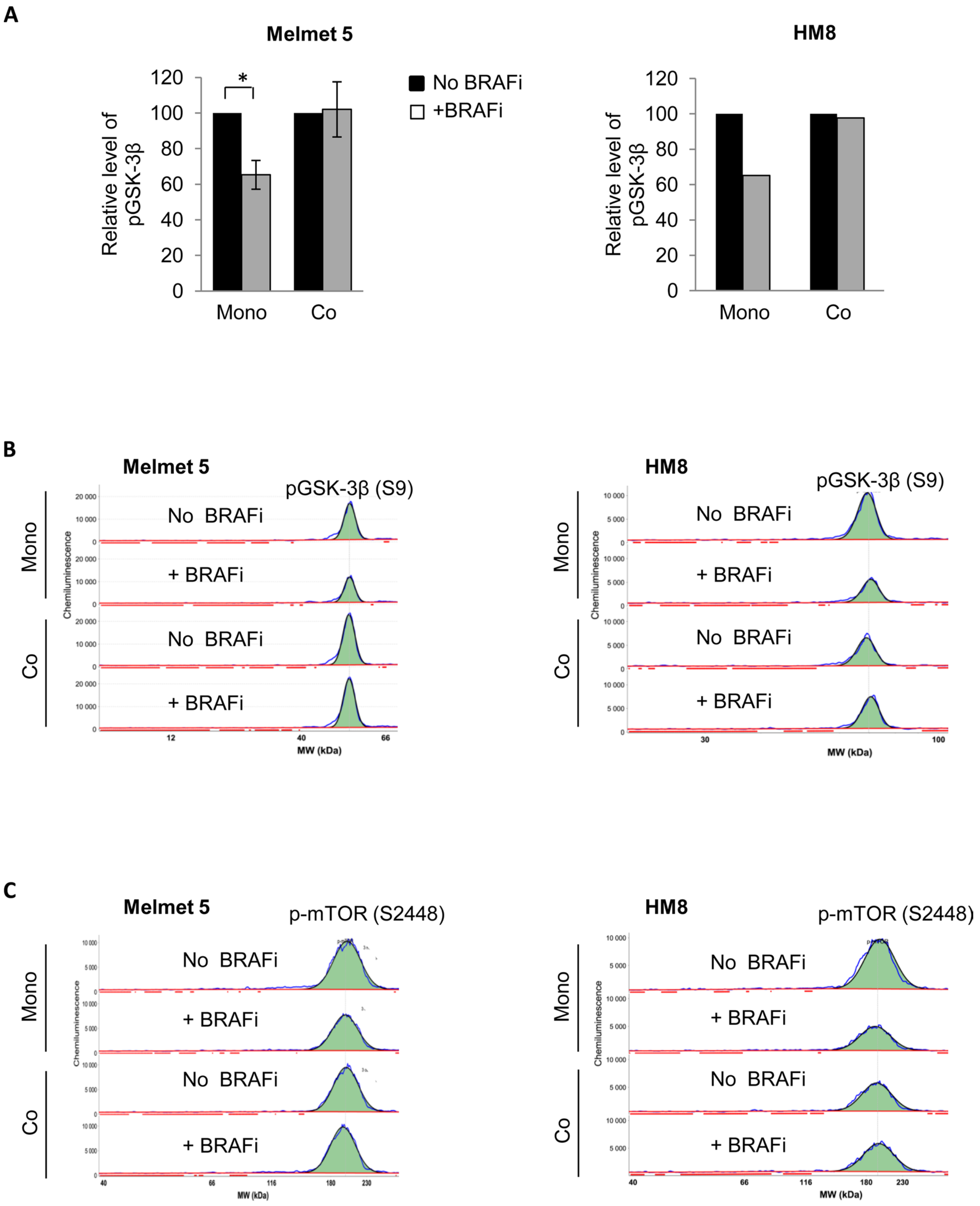

Supplementary Figure S3: The levels of pGSK-3β and p-mTOR as detected by Simple Western immunoassay A. Relative levels of pGSK-3β in Melmet 5 (n=3) and HM8 (n=1) melanoma cells FACS-isolated from mono-cultures or co-cultures with fibroblasts with/without treatment with 1 µM BRAFi for 24 h. The level of pGSK-3β was normalized to the loading control GAPDH (n=3 for Melmet 5 and n=1 for HM8). *, $p<0.05$ (unpaired t-test). **B, C.** Representative peaks for pGSK-3β (B**)** and p-mTOR (C) from automated Simple Western. The protein levels were calculated by integration of the area below peaks (shown in green) detected by chemiluminescence.

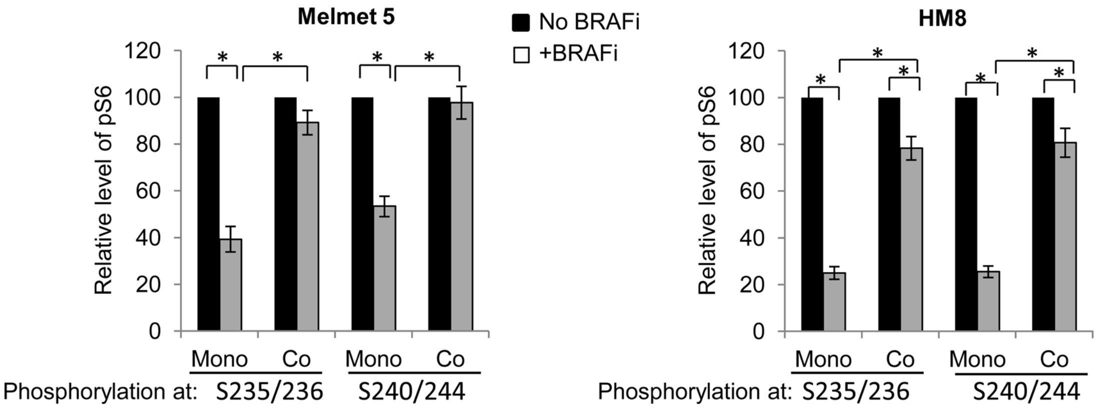

Supplementary Figure S4: The level of S6 phosphorylated at S235/236 site *versus* S240/244 sites as detected by RPPA. Melanoma cells were grown as mono-cultures or co-cultures with fibroblasts, treated with 1 µM BRAFi for 24 h (controls were not treated), isolated by FACS and analyzed by RPPA. The values for the indicated pS6 in the treated samples relative to the respective non-treated controls (set to 100) are shown as average ± SEM (n=3). *, $p<0.05$ (unpaired t-test).

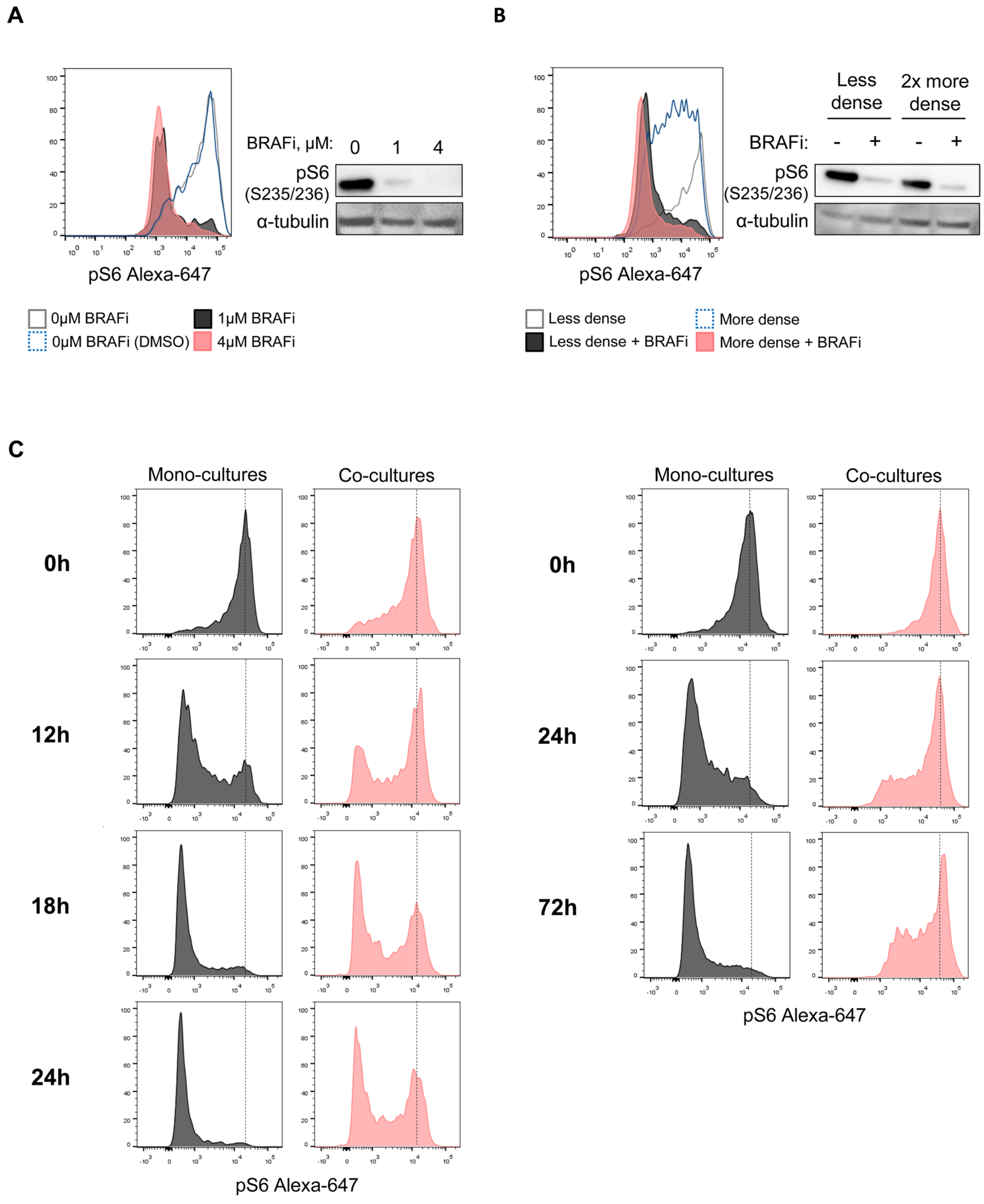

Supplementary Figure S5: Flow cytometric analysis of pS6 levels in melanoma cells under various experimental conditions. A, B. Melmet 5 melanoma cells were grown as mono-cultures and treated with two different doses of BRAFi **A.**, or with 1 μM BRAFi when the cells were at different densities (2-fold difference) **(B).** After 24 h treatment, the samples were analyzed for pS6 by flow cytometry (left) and the data were confirmed by Western blotting (right). **C.** Melmet 5 melanoma cells were grown as mono-cultures or co-cultures with the fibroblasts and treated with 1 μM BRAFi for different durations (controls were not treated) before analysis for pS6 levels by flow cytometry. Histograms from a single experiment are shown; dotted lines designate the histogram peak position in the non-treated cells.

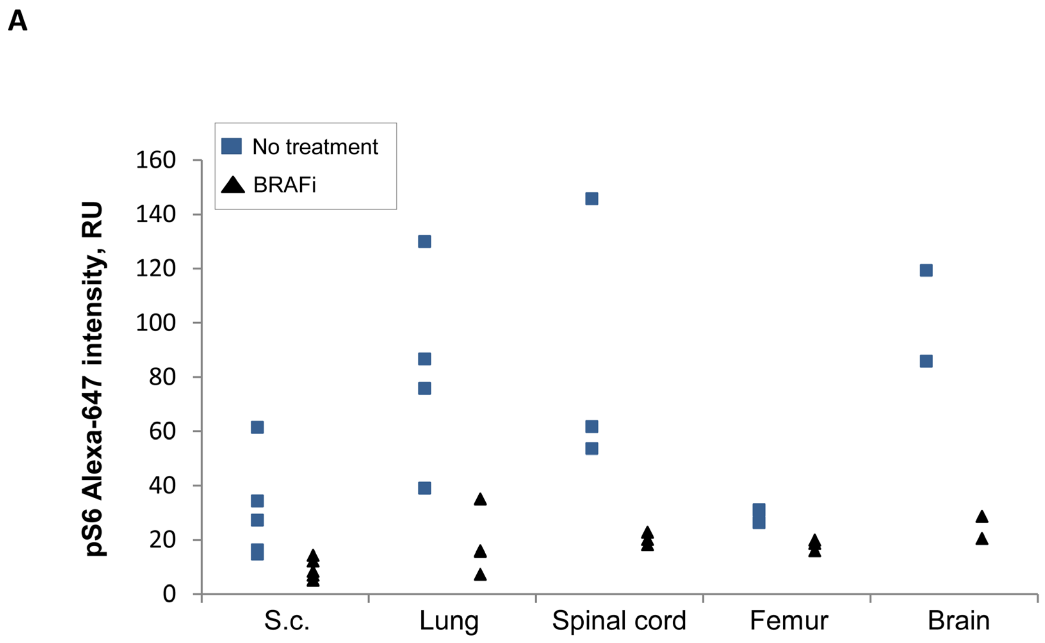

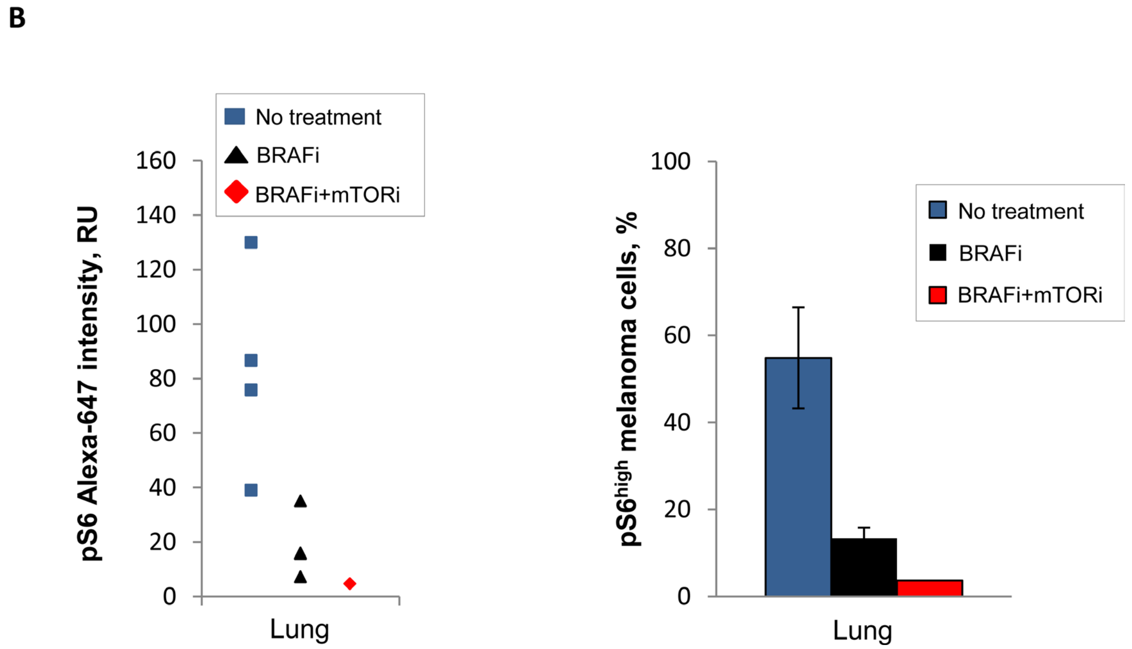

Supplementary Figure S6: Analysis of pS6 in melanoma cells from distinct sites *in vivo* with/without treatment. A. The samples were prepared and analyzed as described in Figure 9A, i.e. Melmet 5 cells were allowed to grow in distinct organs in mice treated with 50 mg/kg BRAFi (five times in total; controls received only vehicle) before the tumors were collected and analyzed for pS6 by flow cytometry. The symbols indicate the mean level of pS6 (shown as relative units (RU)) in single melanoma cells in each sample. The quantification was performed following the strategy illustrated in Supplementary Figure S8C. **B.** The mouse bearing lung metastases was treated with BRAFi as in (A) and additionally, treated with 5 mg/kg mTORi (three times in total). The metastatic lung was analyzed for pS6 as above. The mean level of pS6 (left) and percentage of $pS6^{high}$ melanoma cells (right) after the BRAFi+mTORi combination is shown in red; the data for "No treatment" and "BRAFi" (taken from (A)) are included for comparison. Left, the data for each sample; right, average ± SEM (n=3-4)

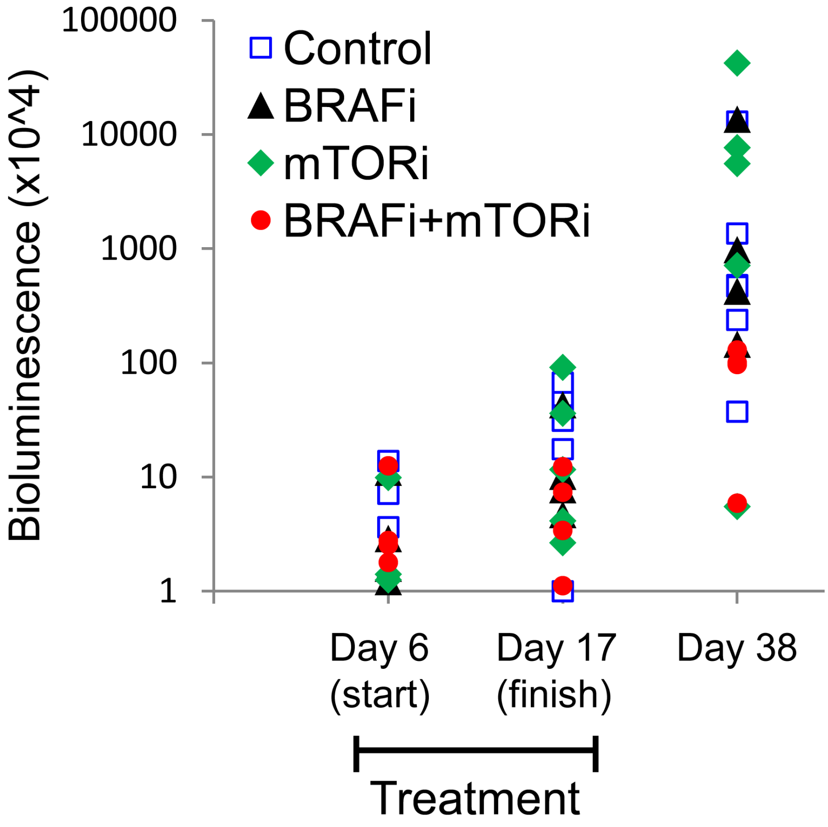

Supplementary Figure S7: Treatment effect on lung metastases *in vivo*: the non-normalized data for each animal. The samples were prepared as in Figure 12, i.e. Melmet 5 cells were injected into mice i.v. and were allowed to grow for 6 days before initiation of the treatment with 50 mg/kg BRAFi, 5 mg/kg mTORi, a combination of both drugs or a vehicle (control) every weekday until day 17, when the treatment was stopped. Tumor burden was scored at days 6 (start), 17 (finish) and 38 (post-treatment) by live imaging *in vivo* measuring bioluminescence (p/s/cm^2/sr). The non-normalized bioluminescence signal intensities in each animal (color-coded for each treatment group) are presented.

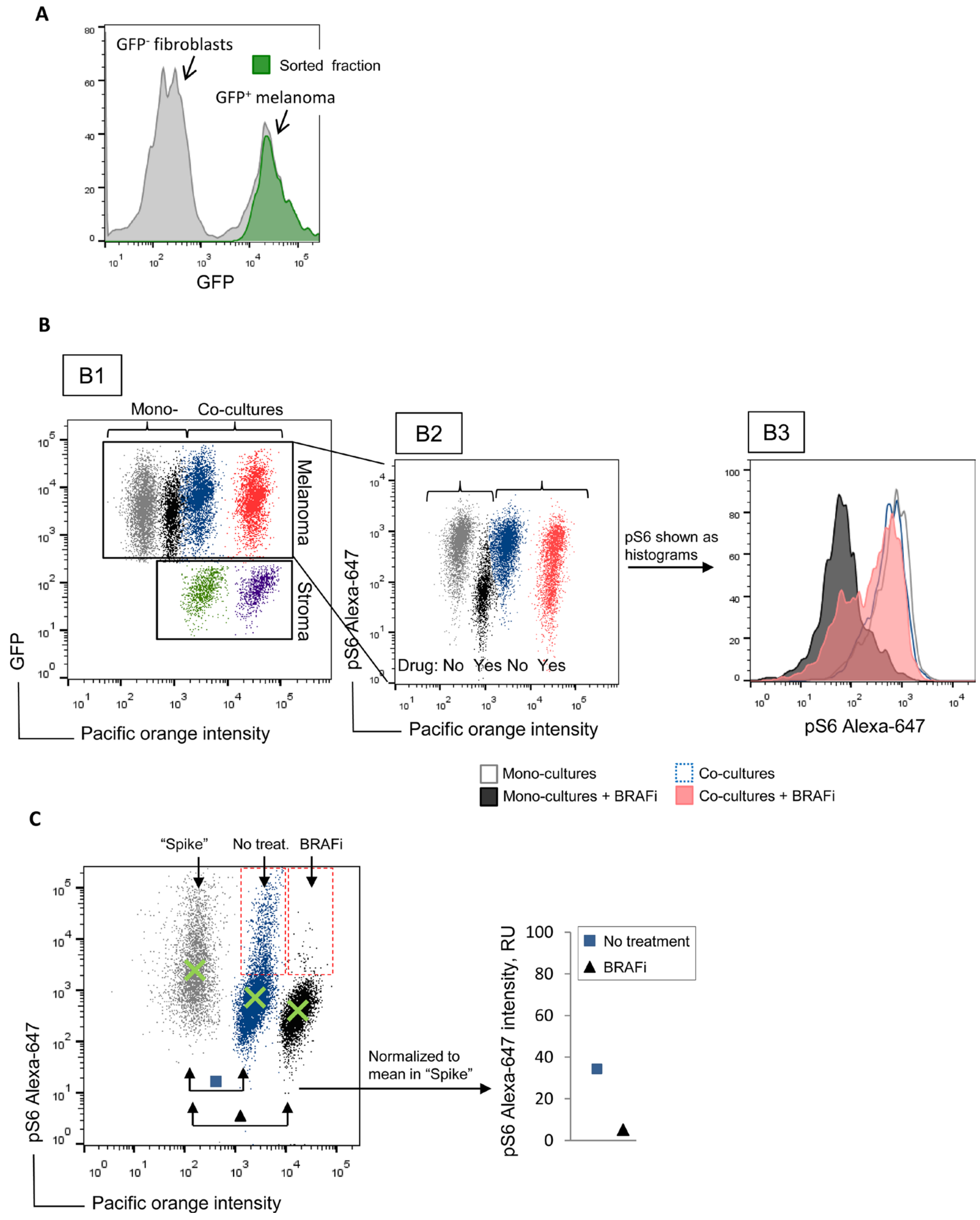

Supplementary Figure S8: A strategy for FACS of GFP+ melanoma cells and flow cytometric analysis of phospho proteins in melanoma cells from *in vitro* and *in vivo*. A. A co-culture sample analyzed for GFP, where two clearly separated peaks – indicating GFP- stromal cells (fibroblasts) and GFP+ melanoma cells – are visible. For FACS separation, a stringent gate was set to collect only clearly GFP-positive melanoma cells (indicated in green). **B.** Four samples: two from GFP+ melanoma mono-cultures (non-treated and treated) and two from co-cultures with GFP- stroma cells (non-treated and treated) were barcoded with increasing concentrations of the dye pacific orange (PO). The barcoded cells were then mixed together and stained with antibodies (Alexa 647-labeled pS6 antibody is shown as example). Based on GFP signal, melanoma cells were distinguished from the stromal cells (B1) and further analyzed for pS6 levels (B2), which eventually were presented as histograms (B3). **C.** Three samples: "spike control" (i.e. Melmet 5 from *in vitro*), non-treated metastatic organ and treated metastatic organ were barcoded with PO, mixed and stained with Alexa 647-labeled pS6 antibody. The analysis was performed as in (B), i.e. first distinguishing GFP+ melanoma cells from the stroma and then calculating Alexa 647 signal mean in each sample (marked by crosses). The signal value in the "spike" was set to 100, and the values in the *in vivo* samples were calculated accordingly and plotted on the Y axis. The percentage of pS6^{high} cells from dot-plots (marked by red boxes) was quantified.
